# Ovarian Reserve in Women With Neuromyelitis Optica Spectrum Disorder

**DOI:** 10.3389/fneur.2018.00446

**Published:** 2018-06-19

**Authors:** Jan Thöne, Solveig Lichtenberg, Anna Stahl, Florence Pache, Ingo Kleiter, Klemens Ruprecht, Ralf Gold, Kerstin Hellwig

**Affiliations:** ^1^Department of Neurology, Katholische Kliniken Ruhrhalbinsel, Essen, Germany; ^2^Department of Neurology, St. Josef Hospital, Ruhr-University Bochum, Bochum, Germany; ^3^Department of Pediatrics, Ruhr-University Bochum, Bochum, Germany; ^4^Department of Neurology, Charité -Universitätsmedizin Berlin, Berlin, Germany

**Keywords:** ovarian reserve, fecundity, childlessness, reproduction, autoimmune diseases of the nervous system

## Abstract

Neuromyelitis optica spectrum disorder (NMOSD) is a neuroinflammatory disease. The majority of NMOSD patients is seropositive for aquaporin-4 (AQP4) antibodies. AQP4 is the main water channel protein in the central nervous system, but has also been identified in the female reproductive system. Fertility issues and ovarian reserve has not yet been studied in females with NMOSD. The purpose of this study was to measure serum Anti-Müllerian hormone (AMH) in females with NMOSD compared to healthy controls (HC), in combination with other lifestyle and reproduction parameters. AMH is independent from the menstrual cycle and a reliable indicator of both ovarian reserve and ovarian function. We included a total of 32 reproductive-age females, 18 HC and 14 with NMOSD. We used an enzymatically amplified two-site immunoassay to determine serum AMH level. In comparison to HC, mean AMH value was reduced in NMOSD. Apart from that significantly more women with NMOSD showed low AMH levels (< 0.8 ng/ml). Low AMH was associated with disease activity. In contrast, none of the immunotherapies for NMOSD, neither any reproductive life style parameter was associated with a decreased AMH. Our results contribute to understanding of hindered fertility in females with NMOSD and enables neurologists to better counsel female patients.

## Introduction

Until detection of a highly specific serum immunoglobulin (Ig)-G autoantibody, neuromyelitis optica spectrum disorder (NMOSD) had previously been considered a multiple sclerosis (MS) variant. The diagnosis depends on recognizing the characteristic clinical picture, magnetic resonance imaging and detection of serum immunoglobulin (Ig)-G autoantibody (NMO-IgG) (1). The primary antigen for NMO-IgG is aquaporin-4 (AQP4), the main water channel protein in the central nervous system (CNS) (2). However, AQP4 has also been identified in a variety of other tissues including kidney, anterior pituitary, and in the reproductive system, to name just a few (3, 4).

Neuromyelitis optica spectrum disorder affects significantly more women than men by a ratio of 9-10/1 in seropositive and 2:1 in seronegative patients usually during their childbearing age, although symptoms can occur at any age from childhood to late adulthood (2, 5).

Anti-Müllerian hormone (AMH), secreted by ovarian granulosa cells, is a homodimeric glycoprotein from the transforming growth factor-beta (TGF-β) superfamily (6). Accumulating evidence has shown that AMH is a good marker of ovarian reserve which describes the ovarian follicle quality and content at any given time. Usually, serum AMH levels in females peak during puberty, remain fairly constant during young adulthood but then bit by bit fall over reproductive time until it decreases below limit of detection at menopause (7, 8). AMH serum values show minimal diurnal or circadian variation and change during the menstrual cycle is low (9). This contrasts with variation in levels of other female sex hormones such as luteinizing hormone (LH), follicle stimulating hormone (FSH), estrogen and progesterone (10). We and others revealed that serum AMH levels are lower in reproductive aged women with immune mediated diseases such as MS and chronic inflammatory rheumatic diseases (10–12). We also found that AMH were lower in MS women not currently treated with immunomodulatory treatment. Furthermore, cytokines important in immune mediated disease may also affect ovarian function (13).

While the impact of pregnancy on NMOSD disease course has been evaluated, less reports assessed the influence of NMOSD on reproductive issues such as fertility, despite the fact that hypothamic lesions are common and therefore might contribute to fertility (14–16). In this study we analyzed serum AMH values in non-pregnant women with NMOSD. We also evaluated the potential contribution of disease modifying agents and disease activity on AMH.

## Materials and methods

### Study population

Serum samples from reproductive-aged women with NMOSD (*n* = 14) were evaluated in a blinded fashion for the presence of serum AMH with a highly specific sandwich ELISA. Other covariates including demographics, body-mass index (BMI), hormonal contraception, age, and nicotine consumption were also collected. As age is the most important confounder for reduced ovarian reserve, we matched our cohort a priori for age. Age-matched healthy female controls (HC, *n* = 18) served as controls. Except oral contraception HC did not have other medications. All participants were of Caucasian origin and reported on a regular menstruation.

Exclusion criteria were: age < 18 years and > 45 years, pregnancy, current lactation, menopause, abnormal thyroid function, chronic liver or autoimmune disease (other than NMOSD), kidney or gynecological disease and therapy with agents toxic to reproduction (16). We also excluded females seeking advice on endocrine dysfunction and impaired fertility.

The protocol was approved by the institutional review board. Patients provided written informed consent before undergoing any procedures. All experiments have been carried out in accordance with *The Code of Ethics of the World Association (Declaration of Helsinki)*. All datasets for this study are included in the manuscript and the Supplementary Files.

### Sample collection and enzyme linked immunosorbent assay (ELISA) experiments

Serum were collected, aliquoted and stored at −20°C. Immediately before measurement, aliquots were brought to room temperature and analyzed for AMH using a sandwich ELISA kit (AMH Gen II ELISA, Beckman Coulter, Webster, USA) according to manufacturers' protocols.

### Statistical analyses

Statistical analyses were performed using the Prism software (GraphPad, San Diego, CA). Data are provided as mean ± SEM. Significances between groups were examined using the Mann-Whitney *U*-test. Other statistical analyses were performed using repeated measurement ANOVA for multiple comparisons. In all experiments, a *p* < 0.05 was defined as statistically significant and *p* < 0.01 was considered as highly statistically significant.

## Results

### Clinical data

We included 14 women with NMOSD and 18 HCs. Characteristics of NMOSD patients are summarized in Table 1. Groups did not differ significantly with respect to age, BMI, use of birth control pill and smoking habits (Supplementary Table 1).

**Table 1:** Characteristics of NMOSD patients (#1–14) and HC (#15–33).

**#**	**Age**	**Onset**	**EDSS**	**No. of relapses**	**Current medication**	**AQP4(x) IgG**	**MOG- IgG**	**AMH ng/ml**	**Birth control**	**Children**
1	25–30	2006	7	29	Tocilizumab	+	–	0.457	–	–
2	40–45	2006	6	2	Teriflunomide	+	–	6.543	–	–
3	40–45	2010	7.5	9	Tocilizumab	+	–	0.827	–	–
4	35–40	2008	5	10	Azathioprin	–	–	0.211	–	–
5	30–35	2007	2.5	11	Rituximab	+	–	0.765	+	–
6	35–40	2013	4	8	DMF	–	–	2.928	+	+
7	20–25	2014	1.5	3	Rituximab	–	+	1.821	+	–
8	20–25	2008	1.5	2	Azathioprin	+	–	3.538	+	+
9	20–25	2015	2	2	Rituximab	+	–	1.456	+	–
10	30–35	2011	2.5	1	Rituximab	+	–	1.335	–	–
11	15–20	2010	3.5	5	Azathioprin	+	–	4.235	–	–
12	40–45	2014	1	1	Rituximab	+	–	0.511	–	–
13	40–45	2010	1	8	Azathioprin	+	–	2.187	–	+
14	35–40	2009	2.5	4	Azathioprin	+	–	2.940	–	–
15	30–35	x	x	x	None	x	x	3.779	+	+
16	40–45	x	x	x	None	x	x	1.191	–	–
17	40–45	x	x	x	None	x	x	0.892	–	+
18	35–40	x	x	x	None	x	x	0.648	–	–
19	30–35	x	x	x	None	x	x	1.875	–	–
20	40–45	x	x	x	None	x	x	0.909	–	–
21	20–25	x	x	x	None	x	x	4.936	+	–
22	20–25	x	x	x	None	x	x	4.901	+	–
23	30–35	x	x	x	None	x	x	4.304	–	–
24	30–35	x	x	x	None	x	x	6.394	–	–
25	20–25	x	x	x	None	x	x	3.922	+	–
26	30–35	x	x	x	None	x	x	1.496	+	–
27	30–35	x	x	x	None	x	x	2.418	–	–
28	40–45	x	x	x	None	x	x	0.929	–	–
29	25–30	x	x	x	None	x	x	7.057	+	–
30	30–35	x	x	x	None	x	x	5.623	+	+
31	20–25	x	x	x	None	x	x	2.461	+	–
32	25–30	x	x	x	None	x	x	4.757	+	–

*In order to protect patients identify and in accordance with journal regulations the patient's age is presented as a range. Dimethyl fumarate (DMF)*.

### Reproductive and gynecological history

The age at menarche did not differ between groups and only 3 participants reported on an irregular menstrual period (Supplementary Table 1). In the NMOSD group, 3 patients (21.4%) had a total of 6 children compared to 3 HCs (16.6%) with a total of 5 children. With one exception females remembered the interval after contraception has been discontinued and the validation of pregnancy. The weeks to pregnancy did not differ between both groups (Supplementary Table 1). No woman had a history of assisted reproductive intervention or miscarriages.

### NMOSD disease specific characteristics

Median EDSS was 3.5 (range 1.0–7.5). Median disease duration was six years (range 1–10). All females with NMOSD were currently treated. The majority received either rituximab (*n* = 5, 35.7%) or azathioprine (*n* = 5, 35.7%) followed by tocilizumab (*n* = 2; 28.6%), teriflunomide (*n* = 1, 7.1%) and dimethyl fumarate (*n* = 1; 7.1%). Antibodies to AQP4 were found in eleven women (78.6%) with NMOSD. One AQP4-IgG-seronegative NMOSD patient (7.1%) was seropositive for myelin oligodendrocyte glycoprotein antibodies (MOG-IgG). Two NMOSD patients (14.2%) were seronegative for both AQP4-IgG and MOG-IgG. No other antibodies were reported in all NMOSD patients.

### Serum AMH

**Figure 1:**
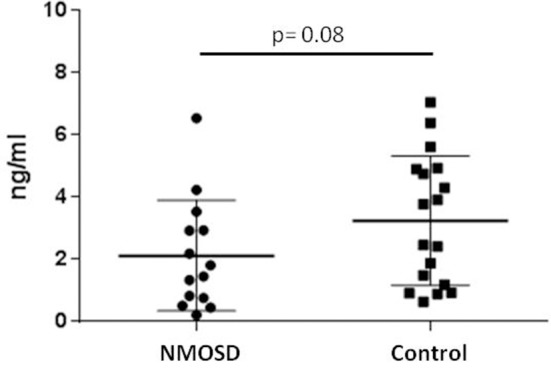
Anti-Müllerian Hormone (AMH) ELISA: NMOSD subjects show reduced mean AMH serum level compared to healthy controls (Mann–Whitney *U-*Test).

The mean AMH level was 2.125 ± 0.47 ng/ml in the NMOSD group compared to 3.250 ± 0.48 ng/ml in HCs (Figure 1, Supplementary Table 2). Next, AMH was categorized in biological meaningful categories (< 0.8 ng/ml = low AMH; < 0.4 ng/ml = very low AMH). Significantly more females with NMOSD (*n* = 4, 28.6%) had AMH levels < 0.8 ng/ml in comparison to HC (*n* = 1, 5.5%). We observed very low AMH values only in one NMOSD patient (7.1%) (Table 1). All reproductive life style factors were not associated with an AMH level < 0.8 ng/ml in the bivariate analysis, neither were NMOSD immune treatments. There was a non-significant difference in EDSS severity between women with low and normal AMH (EDSS: 4.1 ± 1.1 vs. 3.2 ± 0.6; *p* = 0.472). Additionally, females with low AMH reported on more relapses compared to females with normal AMH (12.75 ± 5.8 vs. 5.1 ± 1.0; *p* = 0.193). Neither disease duration (years: 5.0 ± 2.3 vs. 4.3 ± 2.4) nor age at sample collection (35.2 ± 5.6 vs. 32.4 ± 9.6) differed significantly between females with low AMH and normal AMH.

## Discussion

This is the first case series showing that women with NMOSD have lower mean /median AMH levels than healthy controls. We also noted that biological meaningful low levels of AMH (< 0.8 ng/ml) were significantly more frequent in women with NMOSD, consistent with recent studies in females with MS (10, 11). Low AMH was not associated with treatment or other investigated factors.

Our data indicate that ovarian reserve may be impaired in a fraction of female NMOSD patients although the biological reasons remain yet questionable.

An increasing number of women delay childbearing and often numerous years of contraception may precede the plan to conceive. But the size of ovarian reserve and consequently fertility declines throughout life (17). Whereas 1 in 10 women has trouble becoming pregnant 50% of women aged 40–44 report on difficulties becoming pregnant (18); Centers for disease control and prevention, 2013^1^. In our case series patients were age matched and therefore age differences as the strongest confounder for a reduced ovarian reserve cannot explain our results. Apart from that women have to face potential long-term side effects of immunotherapies, including potential unfavorable effects on fertility, and fecundity.

The cause and significance of AMH decrease in NMOSD females remains yet unclear. Ovulation is a complex process on a monthly basis orchestrated by a balanced interaction between various sex steroids. This process can be disturbed both on a local immunological aberrations and on a neuroendocrine level.

AQP4 has been identified in different tissues outside of the CNS but was not found in the human ovary including stroma, cortex and follicles, suggesting that NMO-IgG dependent inflammatory processes of the ovaries and following diminished ovarian reserve is implausible (3). Thyroid function was inconspicuous in all participants. Finally, neither certain NMOSD therapies nor any reproductive lifestyle parameter was associated with low AMH. Similar findings were reported in women with MS (10, 11).

Taken these aspects into account an impairment of the hypothalamic-pituitary-ovary axis (HPOA) seems to be the most attractive cause of AMH decrease and poorer ovarian reserve. Previous studies in AQP4 knockout mice demonstrated disrupted secretion of hormones from the HPOA and subfertility (4, 19). Interestingly, before puberty the appearance of ovaries of AQP4 knockout mice was similar to control mice, but at maturation, the ovaries of knockout mice revealed a marked reduction in follicular maturation (4).

The mammalian ovary has a defined supply of oocytes. Female that enter puberty with a diminished number of oocytes are susceptible of a faster reproductive decline, as their ovarian follicle content is expected to decline faster. Poorer ovarian reserve does not necessarily compromise fecundity and here, pregnancy rates did not differ between groups. Nevertheless, poor ovarian reserve may lead to a premature menopause and subsequent risk of childlessness. Indeed, AMH knockout mice are characterized by a faster depletion of their primordial follicle pool compared to control animals (20). Furthermore, very recently (21) reported on a decreased number of pregnancies and a notable rate of fertility treatments in women with NMOSD (21).

Our study is limited by the small sample size, the etiologic heterogeneity of the NMOSD patients included here and the cross-sectional design, hence, we cannot establish an actual temporal sequence to prove causality. Another potential draw-back is the exclusion of known comorbidities and the inclusion of patients on immunotherapies that may both influence fertility as well. Therefore, we can not survey the real effect of NMOSD on reproductive history. One strength of our study is that this is, to our knowledge, the first examination of AMH in NMOSD females as a marker of ovarian reserve and the classification of AMH in biological significant categories. Our data suggest that females with NMOSD evidence might show a modified endocrine pattern and provide the motivation for future research. Additionally, our data enables neurologists to better advise women with NMOSD with the plan to conceive.

In conclusion, we have shown for the first time that in women with NMOSD serum AMH, an indirect marker of ovarian reserve, is reduced compared to healthy controls. Our data reveal further evidence of a modified endocrine pattern in some women with NMOSD and provide the rationale for future studies.

## Ethics statement

All participants gave written informed consent. The study protocol was approved by the Institutional Ethical Committee of the Ruhr University Bochum, Germany.

## Author contributions

JT devised research, collected data, performed the statistical analysis, and wrote the manuscript. SL, FP, and AS collected data. KH conceived study, designed research, analyzed data, and wrote sections of the manuscript. IK conceived study, designed research, and wrote sections of the manuscript. RG and KR conceived study, designed research. All authors contributed to manuscript revision, read and approved the submitted version.

### Conflict of interest statement

KH, IK, and KR are member of a German-wide network of NMO researchers and clinical neurologists; Neuromyelitis optica study group (NEMOS). KH is supported by a research grant of the German Research Foundation (He 6841/1-1) and has received speaker and research Honoria from Bayer Healthcare, Biogen Idec, Teva Pharmaceutical Industries Ltd, Merck Serono, Sanofi Aventis and Novartis Pharma. IK received honoraria for consultancy or speaking and travel reimbursement from Bayer Healthcare, Biogen, Chugai, Merck, Roche, and Shire, and research support from Affectis, Biogen, Chugai and Diamed, all not related to the presented work. RG reported serving on scientific advisory boards for Teva Pharmaceutical Industries Ltd, Biogen Idec, Bayer Schering Pharma, and Novartis; receiving speaker honoraria from Biogen Idec, Teva Pharmaceutical Industries Ltd, Bayer Schering Pharma, and Novartis; and receiving research support from Teva Pharmaceutical Industries Ltd, Biogen Idec, Bayer Schering Pharma, Merck Serono, and Novartis. KR received grants from German Ministry of Education and Research (BMBF/KKNMS, Competence Network Multiple Sclerosis), Novartis, Merck Serono and the Charite Research Fund; honoraria for consultancy or speaking and travel reimbursement from Novartis, Bayer Healthcare, Biogen Idec, Merck Serono, sanofi-aventis/Genzyme, Teva Pharmaceuticals, and Guthy Jackson Charitable Foundation; all unrelated to the submitted work. The remaining authors declare that the research was conducted in the absence of any commercial or financial relationships that could be construed as a potential conflict of interest.

## Footnotes

^1^https://www.cdc.gov/nchs/fastats/infertility.htm (Accessed July 8, 2013).
