# Supplementary material for: Ovarian Reserve in Women With Neuromyelitis Optica Spectrum Disorder
Source: Front Neurol. 2018 Jun 19;9:446. doi: 10.3389/fneur.2018.00446 (PMC6020788; doi:10.3389/fneur.2018.00446)
Supplement: Supplementary file 1 [file Table_1.DOCX]

**Supplementary Table 1:** Subject characteristics

Control NMOSD p-value

Number of patients, n 18 14

Mean age, years 32.4±6.9 33.2±10.8 >0.05

Age range, years 20 - 42 19 - 42 >0.05

Nicotine consumption, % 38.8 35.7 >0.05

Mean age at menarche, years 12.8±1.0 12.9±1.6 >0.05

Regular menstruation, % 92.8 88.8 >0.05

Use of birth control pill, % 50.0 35.7 >0.05

Patients with children, number 3 3 >0.05

Total number of children 5 6 >0.05

Weeks to pregnancy 15.2±6.5 14.6±2.8 >0.05

Patients with miscarriage 0 0 >0.05

Assisted reproductive intervention 0 0 >0.05

Body Mass Index 22.9±3.6 24.7±4.4 >0.05

|  |  |  |
| --- | --- | --- |
|  |  |  |
