# Supplementary material for: Ovarian Reserve in Women With Neuromyelitis Optica Spectrum Disorder
Source: Front Neurol. 2018 Jun 19;9:446. doi: 10.3389/fneur.2018.00446 (PMC6020788; doi:10.3389/fneur.2018.00446)
Supplement: Supplementary file 2 [file Table_2.DOCX]

Supplementary Table 2: AMH serum level
